# Potential impact of high-density lipoprotein cholesterol in the postoperative outcomes of chronic subdural hematoma patients: multi-institutional study in Korea

**DOI:** 10.1186/s12944-023-01970-5

**Published:** 2023-11-17

**Authors:** Jin Eun, Stephen Ahn, Min Ho Lee, Jin-Gyu Choi, Young Il Kim, Chul Bum Cho, Jae-Sung Park

**Affiliations:** 1 Department of Neurosurgery, Eunpyeong St. Mary’s Hospital, College of Medicine, The Catholic University of Korea, Seoul, Republic of Korea; 2 Department of Neurosurgery, Seoul St. Mary’s Hospital, College of Medicine, The Catholic University of Korea, Seoul, Republic of Korea; 3 Department of Neurosurgery, Uijeongbu St. Mary’s Hospital, College of Medicine, The Catholic University of Korea, Seoul, Republic of Korea; 4 Department of Neurosurgery, Yeouido St. Mary’s Hospital, College of Medicine, The Catholic University of Korea, Seoul, Republic of Korea; 5 Department of Neurosurgery, St. Vincent’s Hospital, College of Medicine, The Catholic University of Korea, Seoul, Republic of Korea

**Keywords:** Chronic subdural hematoma, Burr hole trephination, Cholesterol, Statin

## Abstract

**Background:**

Chronic subdural hematoma (CSDH) is a common clinical situation in neurosurgical practice, but the optimal treatment option is controversial. This study aimed to evaluate the effect of cholesterol-lowering medications on and how they affected the prognoses of CSDH patients.

**Methods:**

In this multi-institutional observational study performed in Korea, data from recently treated CSDH patients were gathered from 5 hospitals. A total of 462 patients were collected from March 2010 to June 2021. Patient clinical characteristics, history of underlying diseases and their treatments, radiologic features, and surgical outcomes were analyzed.

**Results:**

Seventy-five patients experienced recurrences, and 62 had reoperations after the initial burr hole surgery. Among these, 15 patients with recurrences and 12 with reoperations were taking cholesterol-lowering medications. However, the use of medications did not significantly affect recurrence or reoperation rates (*P* = 0.350, *P* = 0.336, respectively). When analyzed by type of medication, no clinically relevant differences in total cholesterol (TC), triglyceride (TG), or low-density lipoprotein cholesterol (LDL-C) levels were identified. The combination of a statin drug and ezetimibe significantly elevated high-density lipoprotein cholesterol (HDL-C) levels (*P* = 0.004). TC, LDL-C, and TG levels did not significantly affect patient prognoses. However, HDL-C levels and recurrence (odds ratio (OR) = 0.96; 95% confidence interval (CI): 0.94–0.99; *p* = 0.010) were negatively correlated. An HDL-C level of 42.50 mg/dL was identified as the threshold for recurrence and reoperation.

**Conclusions:**

In this study, using cholesterol-lowering medications did not significantly impact the prognosis of patients who underwent surgical management for a chronic subdural hematoma. However, the findings showed that the higher the HDL-C level, the lower the probability of recurrence and reoperation.

## Background

A chronic subdural hematoma (CSDH) is mostly caused by trauma. However, unlike an acute subdural hemorrhage, it is influenced by various processes, such as osmotic changes and inflammation [1, 2]. Blocking inflammation and immature angiogenesis with atorvastatin in a rat model led to rapid hematoma absorption [3, 4]. Hematoma drainage using burr hole trephination is an efficient method for reducing hematoma volume. However, the reported recurrence rate is as high as 21%, even with successful surgery [5–9]. Although surgical treatment is the mainstay treatment, research is being conducted on less-invasive treatment options, such as middle meningeal artery (MMA) embolization [10, 11] or the use of certain medications [2, 12–20].

Among the non-surgical treatment interventions for CSDH patients, a randomized controlled trial (RCT) demonstrated the beneficial effects of atorvastatin [16]. Statins (β-hydroxy β-methyl-glutaryl-CoA reductase inhibitors) were tested as a conservative method for treating CSDH patients because of their effects on reducing inflammation in the vessel wall [21] and mobilizing endothelial progenitor cells for vascular repair [22–25]. Based on an RCT that showed the use of atorvastatin reduced the volume of CSDH without surgical intervention, [16] the authors aimed to investigate whether the use of statins would affect recurrence and reoperation in patients who underwent burr hole trephination for a CSDH. This would be the largest published study on the effect of statins on the outcome of CSDH patients.

## Methods

A retrospective analysis was conducted on a total of 462 patients who underwent burr hole trephination for a CSDH from March 2010 to June 2021 at 5 hospitals. The study investigated patient age, sex, the timing of surgery, and medical history of hypertension, diabetes, hyperlipidemia, liver disease, kidney disease, stroke, cardiovascular disease, and hematologic disorders. Lipid profiles, including total cholesterol (TC), high-density lipoprotein cholesterol (HDL-C), low-density lipoprotein cholesterol (LDL-C), and triglyceride (TG) levels, were analyzed, and the type and dose of medication were also collected. Every laboratory value, including LDL-C levels, was measured directly. All patients included in the study were followed for longer than 6 months to assess recurrence and reoperation. The time frame was set up to 6 months following a previous RCT that reported good functional outcomes after the treatment of CSDH patients [26]. An increase in hematoma volume during follow-up was defined as a recurrence, and a volume increase that required additional surgery was defined as a reoperation. Patients already taking a cholesterol-lowering medication on initial presentation were considered the medication group, and patients without cholesterol-lowering medication constituted the control group.

### Statistical methods

For continuous variables, the authors examined the 1st to the 3rd quartile based on the median value. Percentiles were used to assess the proportion of patients with medical histories of other conditions compared to the overall patient population. Linear regression analysis was used to investigate the relationship between the intake of cholesterol-lowering medications and TC, HDL-C, LDL-C, and TG levels. Logistic regression analysis was used to examine the association between lipid levels and the occurrence of reoperation or recurrence. The odds ratio (OR) was used to determine the impact of these factors on prognoses of CSDH patients. Regression analysis was used to assess the differences in cholesterol levels between groups taking cholesterol-lowering medications and the effect of atorvastatin on cholesterol levels. HDL-C threshold levels were assessed using a receiver operating characteristics (ROC) curve. Finally, correlation analysis was performed to examine the influence of each medication on cholesterol levels. Statistical analyses were conducted using R studio, version 4.2.2, by the R Foundation for Statistical Computing in Vienna, Austria. A two-sided *P*-value of 0.05 was considered statistically significant.

## Results

Table 1 describes the baseline characteristics of the patients. The median age of all patients was 75 years (range, 67–81 years), and the medication group was older than the control group (76 vs. 64 years; *P* = 0.003). Fewer male patients were included in the medication group than in the control group (55.36% vs. 71.14%; *P* = 0.003). A history of hypertension was identified in 74.11% of the patients in the medication group and 51.14% of the control group (*P* < 0.001). A history of diabetes mellitus was identified in 45.54% of the patients in the medication group and 25.43% of the control group (*P* < 0.001). Sixty-three patients took cholesterol-lowering medication due to hyperlipidemia, and 49 patients took cholesterol-lowering medication for the primary prevention of stroke, according to their medical histories. The history of liver disease, kidney disease, cancer, and hematologic disease did not differ between the medication group and the control group (*P* = 1.000, *P* = 0.469, *P* = 0.191, and *P* = 0.463, respectively). More patients had ischemic and/or hemorrhagic stroke in the medication group than in the control group (*P* = 0.005), and heart disease was also found more frequently in the medication group (*P* = 0.009).

**Table 1:** Baseline characteristics

	All (n = 462)	Medication group (n = 112)	Control group (n = 350)	*P*-value
Age (median, 1Q-3Q)	75 (67–81)	76 (71–81)	74 (65–81)	0.003*
Sex (%) Male Female	311 (67.32) 151 (32.68)	62 (55.36) 50 (44.64)	249 (71.14) 101 (28.86)	0.003*
CSDH side (%) Both Right Left	78 (16.88) 183 (39.61) 201 (43.51)	24 (21.43) 50 (44.64) 38 (33.93)	54 (15.43) 133 (38.00) 163 (46.57)	0.049*
PAST HISTORY				
HTN (%)	262 (56.71)	83 (74.11)	179 (51.14)	< 0.001*
DM (%)	140 (30.30)	51 (45.54)	89 (25.43)	< 0.001*
HL (%)	84 (18.18)	63 (56.25)	21 (6.00)	< 0.001*
LD (%)	28 (6.06)	7 (6.25)	21 (6.00)	1.000
KD (%)	24 (5.19)	4 (3.57)	20 (5.71)	0.469
CVA (%) Ischemic Hemorrhagic Both	37 (8.01) 3 (0.06) 1 (0.22)	11 (9.82) 3 (2.68) 1 (0.89)	26 (7.43) 0 (0.00) 0 (0.00)	0.005*
CHD (%)	50 (10.82)	20 (17.86)	30 (8.57)	0.009*
Cancer (%)	31 (6.71)	4 (3.57)	27 (7.71)	0.191
HD (%)	10 (2.16)	1 (0.89)	9 (2.57)	0.463
LIPID PROFILE				
TC	152.0 (131.2–178.0)	141.0 (117.5–161.5)	159.0 (137.5–187.5)	0.002*
HDL-C	47.00 (38.00–56.00)	47.00 (38.25–58.25)	46.00 (38.00–56.00)	0.508
LDL-C	87.00 (71.75–108.00)	72.00 (56.50–89.00)	92.00 (76.00–112.00)	< 0.001*
TG	94.0 (72.0–139.2)	97.5 (72.00–136.00)	96.5 (72.0–141.2)	0.140
COMPLICATIONS				
Recurrence	74 (16.02)	15 (13.39)	60 (17.14)	0.381
Reoperation	62 (13.42)	12 (10.71)	50 (14.29)	0.426
Morbidity	15 (3.25)	4 (3.57)	11 (3.14)	0.767
Mortality	12 (2.60)	0 (0.00)	12 (3.43)	0.045*

1Q: 1st quartile, 3Q: 3rd quartiles, CSDH: chronic subdural hematoma, HTN: hypertension, DM: diabetes mellitus, HL: hyperlipidemia, LD: liver disease, KD: kidney disease, CVA: cerebrovascular accident, CHD: coronary heart disease, HD: hematologic disease, TC: total cholesterol, HDL-C: high-density lipoprotein cholesterol, LDL-C: low-density lipoprotein cholesterol, TG: triglycerides; *P-value < 0.05

Cholesterol levels in the patients included a median TC level of 152.0 mg/dL (131.20–178.00 mg/dL), a median HDL-C level of 47.0 mg/dL (38.00–56.00 mg/dL), a median LDL-C level of 87.0 mg/dL (71.75–108.00 mg/dL), and a median TG level of 94.0 mg/dL (72.00–139.20 mg/dL). In the medication group, the median TC level was 141.0 mg/dL (117.50–161.50 mg/dL), the median HDL-C level was 47.0 mg/dL (38.25–58.25 mg/dL), the median LDL-C level was 72.0 mg/dL (56.50–89.00 mg/dL), and median TG level was 97.5 mg/dL (72.00–136.00 mg/dL). In the control group, the median TC level was 159.0 mg/dL (137.50–187.50 mg/dL), the median HDL-C level was 46.00 mg/dL (38.00–56.00 mg/dL), the median LDL-C level was 92.0 mg/dL (76.00–112.00 mg/dL), and the median TG level was 96.5 mg/dL (72.00–141.20 mg/dL). No significant difference in TG levels was found between the medication group and the control group (*P* = 0.140). However, LDL-C and TC levels were significantly lower in the medication group (*P* < 0.001 and *P* = 0.002, respectively). HDL-C levels were not significantly different (*P* = 0.508).

Recurrence occurred in 16.02% and 13.39% of the medication and control groups, respectively (*P* = 0.381). Reoperation was performed in 13.42% of the total patients, and the proportion did not differ between the medication and control groups (10.71% vs. 14.29%; *P* = 0.426). Morbidity, including postoperative epidural hemorrhage, subarachnoid hemorrhage, and kidney injury, occurred in 3.25% of all patients, with 3.57% in the medication group and 3.14% in the control group (*P* = 0.767). No mortality was observed in the medication group, but 2.60% mortality was observed in the control group (*P* = 0.045).

**Fig. 1:**
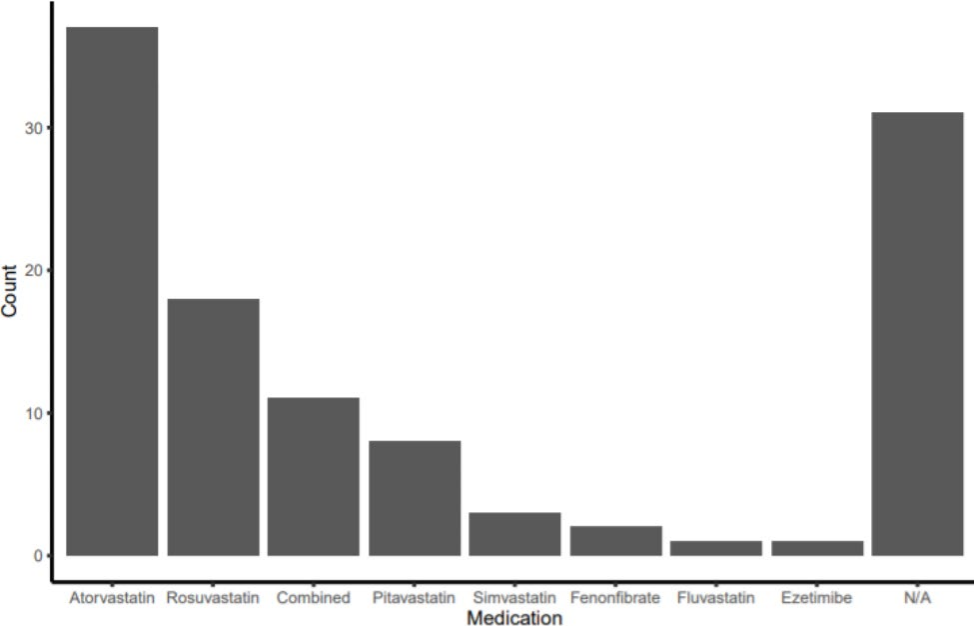
Graph depicting the number of patients taking each cholesterol-lowering medication. N/A: not available

One hundred and twelve patients were found to be taking cholesterol-lowering medication. Of these patients, 37 were taking atorvastatin, 18 were taking rosuvastatin, 8 were taking pitavastatin, 3 were taking simvastatin, one was taking fluvastatin, one was taking only ezetimibe, and 2 were taking fenofibrate. Eleven were taking a combination of ezetimibe and a statin drug. The medication type could not be confirmed in 31 cases (Fig. 1). An comparative analysis was conducted to identify differences in lipid levels among the medication types. There were no significant differences in TC and TG levels, but patients on combined medications showed elevated HDL-C levels (*P* = 0.004). Other medications were not associated with significant differences in HDL-C levels. Patients on pitavastatin showed higher LDL-C levels compared to other patients (*P* = 0.012) (Table 2).

**Table 2:** Cholesterol-lowering medications used and their effects on cholesterol levels (*P*-value)

	Number of patients	TC	HDL-C	LDL-C	TG
Atorvastatin	37	0.382	0.993	0.378	0.418
Rosuvastatin	18	0.723	0.698	0.534	0.646
Combined	11	0.524	0.004*	0.187	0.548
Pitavastatin	8	0.053	0.984	0.012*	0.617
Simvastatin	3	0.994	0.759	0.321	0.660
Fenofibrate	2	0.882	0.244	0.406	0.828
Ezetimibe	1	0.767	0.495	N/A	0.739
Fluvastatin	1	N/A	N/A	N/A	N/A
N/A	31	0.217	0.282	0.379	0.088
Total	112				

TC: total cholesterol, HDL-C: high-density lipoprotein cholesterol, LDL-C: low-density lipoprotein cholesterol, TG: triglycerides, N/A: not available; *P-value < 0.05

Further analysis of patients taking atorvastatin was conducted for comparison to previous studies. The median TC level in patients taking atorvastatin was 137.0 mg/dL (115.00–162.00 mg/dL), the median HDL-C level was 46.0 mg/dL (39.50–61.00 mg/dL), the median LDL-C level was 66.0 mg/dL (56.00–86.00 mg/dL), and the median TG level was 89.5 mg/dL (67.75–113.50 mg/dL). TC and LDL-C levels were significantly lower in patients taking atorvastatin compared to the control group (*P* = 0.006 and *P* < 0.001), but there was no significant impact on TG and HDL-C levels (*P* = 0.205 and *P* = 0.649). Taking atorvastatin did not significantly impact recurrence or reoperation rates (*P* = 0.279 and *P* = 0.451).

**Fig. 2:**
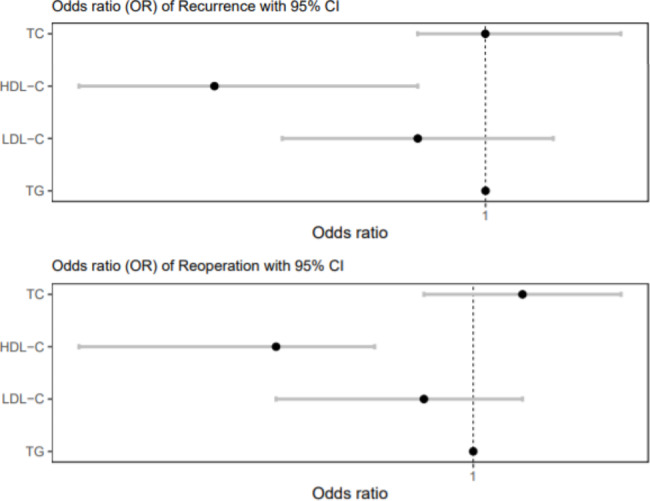
Odds ratios (ORs) of cholesterol for recurrence and reoperation. For recurrence, TC, HDL-C, LDL-C, and TG had ORs of 1.00 (0.99–1.02), 0.96 (0.92–0.99), 0.99 (0.97–1.01), and 1.00 (1.00–1.00), respectively. For reoperation, TC, HDL-C, LDL-C, and TG levels had ORs of 1.01 (0.99–1.03), 0.96 (0.92–0.98), 0.99 (0.96–1.01), and 1.00 (1.00–1.00), respectively. TC: total cholesterol, HDL-C: high-density lipoprotein cholesterol, LDL-C: low-density lipoprotein cholesterol, TG: triglycerides

**Fig. 3:**
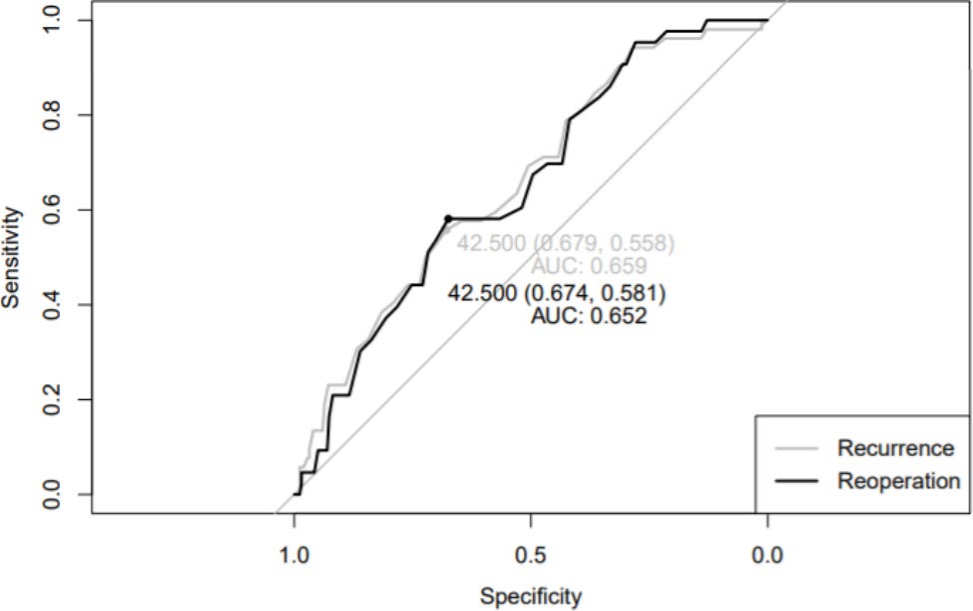
ROC curve analysis of high-density lipoprotein cholesterol levels in patients with chronic subdural hematomas. For an HDL-C threshold level of 42.500 mg/dL the area under the curve (AUC) was 0.659 for recurrence and 0.652 for reoperation

Further analyses were conducted to investigate the effect of serum cholesterol levels on chronic subdural hematomas. In the analysis of the effect on recurrence, TC had an OR of 1.00 (0.99–1.02) and did not affect recurrence (*P* = 0.886). LDL-C levels had an OR of 0.99 (0.97–1.01) (*P* = 0.461), and TG levels had an OR of 1.00 (1.00–1.00), indicating no association with recurrence (*P* = 0.876). However, an OR of 0.96 (0.94–0.99) was found for HDL-C levels, indicating a negative correlation, where an increase in HDL-C levels was associated with a decrease in recurrence rates (*P* = 0.010). Similarly, for reoperation, TC levels had an OR of 1.01 (0.99–1.03) (*P* = 0.656), and LDL-C levels had an OR of 0.99 (0.96–1.01) (*P* = 0.526). TG had an OR of 1.00 (1.00–1.00), indicating no association with reoperation (*P* = 0.831). However, an OR of 0.96 (0.92–0.98) was found for HDL-C levels, indicating that an increase in HDL-C levels was associated with a decrease in reoperation rates (*P* = 0.007) (Table 3; Fig. 2). A threshold of 42.50 mg/dL HDL-C was obtained from the ROC curve (Fig. 3).

**Table 3:** Analysis of the relationship between cholesterol levels and recurrence/reoperation for chronic subdural hematoma

Recurrence	OR (2.5-97.5%)	*P*-value	Reoperation	OR (2.5-97.5%)	*P*-value
TC	1.00 (0.99–1.02)	0.886	**TC**	1.01 (0.99–1.03)	0.656
HDL-C	0.96 (0.94–0.99)	0.010*	**HDL-C**	0.96 (0.92–0.98)	0.007*
LDL-C	0.99 (0.97–1.01)	0.461	**LDL-C**	0.99 (0.96–1.01)	0.526
TG	1.00 (1.00–1.00)	0.876	**TG**	1.00 (1.00–1.00)	0.831

OR: odds ratio, TC: total cholesterol, HDL-C: high-density lipoprotein cholesterol, LDL-C: low-density lipoprotein cholesterol, TG: triglycerides; *P-value < 0.05

## Discussion

Previous studies analyzed the effects of statin medications on the reduction of chronic subdural hematoma volume and recurrence after surgery (Table 4).

**Table 4:** Review of previous articles on the effects of statins in chronic subdural hematoma patients

	Year	Nationality	Study design	Total No.	Subgroup distribution	Treatment measures	Medication dose	Outcomes in the statin group
**Xu M. et al.**	2016	China	Retrospective	109	7 conservative 102 post-surgical : 39 (atorvastatin) vs. 63 (control)	Conservative (with atorvastatin) or post-surgical (with or without atorvastatin)	Atorvastatin 20 mg	Successful treatment of the conservative group More favorable post-surgical outcomes
**Chan D. et al.**	2017	China	Retrospective	24	12 atorvastatin 12 control	Post-surgical patients treated with atorvastatin	Atorvastatin 20 mg	Less risk of deterioration requiring surgery
**Tang R. et al.**	2018	China	Retrospective	245	125 atorvastatin 120 control	Post-surgical patients with or without atorvastatin	Atorvastatin 20 mg	Less recurrence
**Jiang R. et al.**	2018	China	RCT	200	98 patients in each group (atorvastatin or placebo)	Conservative measures with atorvastatin	Atorvastatin 20 mg	More reduction in hematoma volume and superior neurologic function improvement Fewer patients requiring surgery
**Guidry BS. et al.**	2021	USA	Retrospective	111	36 statin 75 no statin	Post-surgical patients with or without statins	NA	Greater reduction in hematoma size
**Klein J. et al.**	2021	Germany	Retrospective	407	123 statin 284 no statin	Post-surgical patients with or without statins	NA	No evidence of a protective effect
**Present study**		Korea	Retrospective	462	112 statin 350 no statin	Post-surgical patients with or without statins	NA	No significant effect on recurrence or reoperation

No.: number of patients analyzed in the article, RCT: randomized controlled trial, NA: not available

The benefits of atorvastatin in treating CSDH have been repeatedly reported since 2016. Xu et al. reported favorable outcomes for patients treated with CSDH in conservative and postoperative settings (*P* = 0.045) [20]. However, only 7 patients were included in the conservative management group, and since these patients had relatively minor bleeding, the outcome may not have differed even without atorvastatin treatment. Chan et al. demonstrated that the risk of deterioration requiring further surgical treatment was lower in the atorvastatin group compared to a Glasgow Coma Scale or Markwalder’s Grading Scale-matched control group (*P* = 0.0447) [12]. However, since only 12 patients were included, further validation is mandatory. In 2018, Tang et al. reported a larger-scale retrospective study in post-surgical CSDH patients treated with atorvastatin. The study found less recurrence compared to the control group [18]. Finally, in 2018, Jiang et al. published the results of an RCT of 20 mg of atorvastatin compared to placebo for the conservative management of CSDH patients [16]. Patients taking atorvastatin showed greater reductions in hematoma volume (*P* = 0.003) and superior neurologic function improvement (*P* = 0.03). Fewer patients required surgery in the atorvastatin group compared to the placebo group (11.2% vs. 23.5%; *P* = 0.03). These CSDH studies on atorvastatin were exclusively performed in China.

Studies that were performed elsewhere were not uniformly treated with atorvastatin. Guidry et al. performed a study in the United States and also reported that statin use was associated with decreases in hematoma size [13]. Unique to this study, no significant difference in outcomes was found according to race. Klein et al. performed a study in Germany, and no evidence for the protective effect of statin medication was found in the treatment of CSDH patients [17]. Similarly, the present study results also showed no significant effect on recurrence or reoperation according to statin usage.

Many of the previously reported studies focused on the usage of atorvastatin medication and the outcome of CSDH patients. Various combinations of cholesterol-lowering drugs are used in daily clinical activities, and this makes interpretations more complex. The protective effect of atorvastatin in post-surgical patients still seems debatable, and evidence that atorvastatin is better than other cholesterol-lowering drugs for treating CSDH patients is scarce. To overcome this hurdle in data analysis, the authors gathered information on the type and dosage of cholesterol-lowering drugs used by the patients. TC, LDL-C, HDL-C, and TG blood chemistry results were collected to better identify which patients truly benefitted from CSDH management.

The results of this study failed to demonstrate the protective effect of statins on the recurrence and reoperation rates of CSDH patients. Even atorvastatin, which had been demonstrated multiple times to be effective, failed to show a significant impact on recurrence or reoperation rates. Notably, the analysis of serum cholesterol levels found that higher levels of HDL-C were associated with a lower incidence of recurrence and reoperation. The cut-off HDL-C value in the study was 42.50 mg/dL. A retrospective single-center study reported by Liu et al. in 2021 also attempted to identify the factors related to CSDH recurrence, including serum lipid levels. They found that CSDH recurrence was affected by risk factors including age, diabetes mellitus, midline shift, and HDL-C levels [27]. The reported an HDL-C cut-off value of 37.45 mg/dL, which was slightly lower than in this report.

Although this study did not find a significant difference in HDL-C levels between patients who used statins and those who did not, differences in HDL-C levels were found to depend on the type of medication used. When the type of medication used and the serum lipid profile levels were analyzed, only patients receiving combined medication with ezetimibe showed elevated HDL-C levels. Ezetimibe reduces atherogenic lipid profiles and increases HDL-C levels, which may explain why HDL-C levels were higher in patients who used a combination of these medications [28]. Even though this study failed to demonstrate it, statins are known to moderately increase HDL-C levels [29].

The anti-inflammatory effect of HDL has been shown in many studies, [30, 31] and a relationship among HDL-C, vascular endothelial growth factor (VEGF), and CSDH was demonstrated. Weigel et al. proposed that VEGF contributes to hematoma growth and CT appearances in CSDH patients [32]. Petrov et al. reported imbalances in angiogenesis factors, including VEGF, in CSDH patients compared to healthy volunteers and that these imbalances were related to rebleeding [33]. A significant reduction in plasma VEGF concentrations was observed following statin therapy in a systemic review published in 2015 [34]. The effect was related to treatment duration, LDL-C lowering activity, the lipophilicity of statins, and the health status of studied individuals but not to the molar dose of statins. Epidemiological and prospective studies proved the vasculoprotective effects of HDL-C, which were presumably by regulating angiogenesis [35]. HDL-C increased endothelial proliferation, migration, and tube formation dose-dependently, consequently promoting angiogenesis via vascular endothelial growth factor 2 activation [36]. The findings in this study that elevated HDL-C levels were related to a lower incidence of CSDH recurrence and reoperation may have been due to the vasculoprotective effect of HDL-C via VEGF-promoting angiogenesis. According to the proposed mechanism mentioned above, there is also a possibility that postoperative CSDH patients may benefit clinically from elevating HDL-C levels with combined cholesterol-lowering medication, including ezetimibe.

### Strengths of the study

This study analyzed the effects of cholesterol-lowering drugs on the outcome of patients with chronic subdural hematoma as a multi-institutional retrospective study. HDL-C levels rather than the usage of statin medication itself were identified to be associated with CSDH patient outcomes. This finding is consistent with the results of previously reported studies, that the impact of cholesterol-lowering drugs on chronic subdural hematoma is still debatable. Instead, patients’ HDL-C levels may be evaluated to better predict their prognoses.

### Limitations

There were several limitations to this study besides its retrospective nature. The study was performed as a multi-center study to provide more diverse patient characteristics and treatment results. However, potential errors or differences in data collection may have been present. Further, the use of other drugs that affect cholesterol mechanisms, such as steroids or retinoids, could not be assessed. The case number was relatively large compared to previously published studies, but quantitative analysis of the dosage of each cholesterol-lowering medication failed to demonstrate statistically relevant results. Further studies with prospective designs are needed to overcome this limitation.

## Conclusions

CSDH patients with elevated HDL-C levels are likely to have superior prognoses in terms of recurrence and reoperation. Thus, patients without elevated HDL-C levels should be carefully assessed during follow-up compared to those with elevated HDL-C levels. Moreover, attempts to elevate patients’ HDL-C levels may facilitate better prognoses.

## Data Availability

Access to the datasets can be provided upon reasonable request the corresponding author.

## List of abbreviations

CSDH: Chronic subdural hematoma
OR: Odds ratio
TC: Total cholesterol
TG: Triglyceride
LDL-C.: Low-density lipoprotein cholesterol
MMA: Middle meningeal artery
RCT: Randomized controlled trial
VEGF: Vascular endothelial growth factor
